# Influence of the Perception of Barriers in Practice of PA in Adolescents: Explanatory Model

**DOI:** 10.3390/healthcare9040380

**Published:** 2021-04-01

**Authors:** Iago Portela-Pino, Myriam Alvariñas-Villaverde, Javier Martínez-Torres, Margarita Pino-Juste

**Affiliations:** 1 Sports Sciences, Universidad Internacional Isabel I. Health Sciences, 09003 Burgos, Spain; 2 Special Didactics, Education and Sport Sciences, Universidad de Vigo, 36005 Pontevedra, Spain; myalva@uvigo.es; 3 Applied Mathematics I, Telecommunications Engineering School, Universidad de Vigo, 36310 Vigo, Spain; javmartinez@uvigo.es; 4 Didactics, School Organization and Research Methods, Education and Sport Sciences, Universidad de Vigo, 36005 Pontevedra, Spain; mpino@uvigo.es

**Keywords:** barriers, PA, predicting variables, sedentarism, NCD

## Abstract

Background: Sedentarism is an important risk factor for non-communicable diseases. To avoid it, it is necessary to establish the barriers which influence a low level of practice of Physical Activity. Methods: This study, conducted with 833 students, aims to describe a model to explain the barriers determining the level of practice of Physical Activity in adolescents according to age, school year, BMI and gender. The inclusion of the analyzed barriers followed the tetra-factorial model: Body image/physical and social anxiety; Tiredness/laziness; Responsibilities/lack of time and Environment/facilities. Results: The barriers to Physical Activity in adolescents are fatigue and sloth, and temporary obligations. The barrier that least influences the practice of Physical Activity is the environment and body image. It is determined that the subjects with the lowest Physical Activity index were those with a high fatigue and laziness score and higher age. The level of physical activity of this population is medium (95% CI, 2.8274–2.9418). Conclusions: It is necessary to overcome tiredness or apathy towards the practice of Physical Activity, especially in those under 16 years of age.

## 1. Introduction

The high indices of obesity suffered by the global population have been verified [1], not only in developed countries but also in those with a lower GDP [2]. Considering the consequences of this situation, it is vitally important to know the barriers experienced by young people that limit the practice of the right levels of physical activity (PA). It is known that those indicating a higher number of barriers have a lower probability of reaching the weekly optimum levels of PA, and consequentially a higher probability of being inactive [3,4]. Along these lines, Tompkins et al. [5] point out that, if parents do not accurately perceive their child’s overweight or obesity status and the potential risk that this situation entails for health, they are usually less inclined to encourage children to adopt healthy behaviors. Being able to overcome these perceived barriers can increase the index of PA [6].

The perception of personal barriers to the practice of PA varies according to the sociodemographic characteristics of the individual [7] and can be dealt with by the use of personal and combined approaches using educational programs, motivational interviews, exercise practice and problem solving [8].

In children, specifically, perceived barriers are the most important predictive factors in a change towards healthy behavior and the most consistent negative correlate of real PA [9].

On the other hand, the suppression at an early age of these barriers is very important because obese adults have a lower probability of practicing a physical exercise than an adult with normal weight. This situation is due to the fact that they experience a higher level of discomfort and anxiety during the exercise sessions [10] and face many barriers to the adoption of a physically active lifestyle [11]. It must also be mentioned that the probability of a person participating in physical activities depends mostly on the benefits of and perceived barriers to being physically active [12].

In the most economically vulnerable areas, from the point of view of the parents, the barriers identified were general, such as financial limitations and lack of time, or specific barriers in a specific country, such as organizational difficulties and unsuitable working environments. In contrast, teachers and community workers added, as a barrier, lack of parental knowledge [13].

Those with limited formal education perceive the lack of social support and resources and fear of injury as barriers to the practice of PA [7].

There are also different perceptions of barriers according to the physical environment in which the person lives. That is, parents from rural zones report significantly higher barriers [14]. A community’s interpersonal and intrapersonal barriers correlate negatively with the support of parents for children’s PA [14]. The study by Taylor et al. [15] indicates that children from urban and suburban neighborhoods of great cities, and in rural zones, report most of the barriers to the practice of PA not according to the characteristics of their environment but according to the size of their community’s population.

In the same way, climate and culture must be analyzed. In Kuwait, a country with many hours of heat and sun, the most common perceived barriers were hot weather (75.9%), work duties (71.21%), laziness (44.3%), lack of time (38.6%), family responsibilities (36.1%) and chronic diseases (33.33%) [16]. Among a group of Iranian women, the most common reported barriers were personal, intrapersonal and environmental factors associated with sports environments, and not setting health as a priority [17].

Regarding gender, lack of energy, time and support were three of the five barriers reported by more than 40% of adolescents, statistically more likely in girls than boys, and older children rather than younger [18]. Female adolescents and those living in the city admit more barriers to the practice of PA, and more students classified as inactive. It is clear that lack of time is the main barrier to the practice of PA and the least active subjects and females are those perceiving more barriers [19].

As stated, with gender itself there are also important factors, specifically, as to whether PA is usually practiced or not. Girls that practice PA value health and the development of the skill more, but those not practicing PA say they would consider PA because of the associated affiliation and social recognition [20]. In the study of Zaragoza et al. [9], girls with a lower level of PA perceived more barriers that those with a high level of activity. Some of these barriers were that they did not like PA, they were not good at PA and sports, they were too lazy to perform PA and it was not safe to practice PA outside.

At the intrapersonal level, girls reported more barriers to the practice of PA than boys [15]. Notably, the parents of girls report a higher percentage of barriers related to security (They do not let me go out alone, it is not safe to be playing in the street, park, patio, etc., I’m afraid of getting injured) [14].

With age, PA gains a special importance, because it is strongly related to health development of maturity [12]. Moreover, PA reduces with age [19] and the barriers change (3,6,7).

In the case of university students, the most commonly reported barriers were personal, social and environmental factors such as time constraints, tiredness, stress, family control, or safety issues [21].

Middle-aged adults perceive less benefits than younger and older adults [12]. Moreover, for the promotion of PA at work three categories of barriers were identified. Barriers can be related to physical, psychological and environmental dimensions. Psychological and environmental barriers were always reported as higher than physical barriers [22].

In the elderly, depressive symptoms are an important barrier to participation in PA [11]. Besides, it was found that health barriers are a partial mediator for functional disability and quality of life in relation to physical health, suggesting that perceived barriers can help to explain the association between functional disability and quality of life in relation to physical health [23].

Furthermore, some interventions have successfully reduced barriers perceived by the patients, can be implemented [24]. It is vitally important to perform such interventions at a young age, because beliefs and experiences forged during childhood manifest during adult life [25].

Hence, the importance of establishing a model to predict the probability of sedentarism as a factor of the barriers found.

Therefore, the aim of our study is to design an explanatory model about the barriers that determine the level of practice of PA and its influence depending on age, gender and grade.

## 2. Materials and Methods

### 2.1. Design

The prospective and observational research design were of an exploratory nature [26]. This method references research situations caused by the researcher without a rigorous control of the extraneous variables to the purposes of the research. Even though it was performed in a real situation before the start of the physical education classes, the previously created groups cannot be modified.

Therefore, it is an accidental sample, being a non-probabilistic sampling with the researcher choosing the subject close to hand, without some special selection criteria, just because they belong to certain school group [27]. Therefore, the students who at the time of the survey were in the educational centers of the Autonomous Community selected for the study were selected.

### 2.2. Participants

A total of 833 students of Compulsory Secondary Education (CSE) and Baccalaureate (BAC) in public schools of the Autonomous Community of Galicia (Spain) participated in this study, using a non-probabilistic and intentional sampling. A total of 49% were men and 51% women. A total of 70.8% attend secondary education and 29.2% Baccalaureate. Their ages go from 12 to 18 (Mean (M) = 14.9; Standard Deviation (SD) = 1.75).

Table 1 shows the frequency distribution according to school year, reaching the conclusion of a uniformity of the sample in that regard.

### 2.3. Instrument

In the study, the Self-Report on Barriers to Exercising—Autoinforme de Barreras para la Práctica del Ejercicio Físico—(ABPEF; [28]) was used, adapted by Niñerola et al. [29]. This version has 17 items on a Likert scale from 0 (a reason unlikely to prevent me from practicing physical exercise in the following weeks) to 10 (a reason very likely to prevent me from practicing physical exercise in the following weeks). Domínguez-Alonso et al. [30] have analyzed the psychometrics of the Self-Report on Barriers to Exercising and report the following factors: body image/physical and social anxiety; tiredness/laziness; responsibilities/lack of time; environment/facilities. In that study the questionnaire received a high reliability (α = 0.86).

To measure PA, the Physical Activity Questionnaire—Adolescents questionnaire (PAQ-A) was used, which records the activity carried out in the last 7 days [31]. The PAQ-A score values range from 1 to 5, from lowest to highest activity.

### 2.4. Variables

The independent variables used in this study match the factors of the scale used because the confirmatory factorial analysis showed an adequate adjustment to the data of a tetrafactorial model [30]. The variables are:

Body image/physical and social anxiety: Body image is the representation of the body that each person creates in their own mind. Physical and social anxiety is the worry about the presentation of that physical appearance to others.

Tiredness/laziness: Lack of willpower, apathy or lack of interest in the practice of physical exercise.

Responsibilities/lack of time: Not having time to practice a sport or physical activities due to a saturation of more important or urgent duties.

Environment/facilities: Lack of resources or places to practice PA.

Among those variables, some have been defined as independent (Scale factors) and others as dependent (PA level) as a method to reach a predictive model to explain them. In this case, the dependent variable is the Level of PA of a quantitative nature, but that has been categorized into three levels, to make the correct application of classification models possible [32,33,34]. These authors classified PAQ scores ≤ 2 as “low activity”, >2 and ≤3 as “moderate activity”, and >3 as “high activity”. Therefore, the total proportion PAQ scores were categorized as 121 (14.53%) low (≤2), 352 (42.26%) moderate (>2 and ≤3) and 360 (43.22%) high activity (>3).

Moreover, to explain the results of the three categories model, the classification tree of active and inactive adolescents was also calculated. That classification follows the model by Benítez-Porres et al. [35], setting the discriminant value between classes as 2.75.

### 2.5. Procedure

The questionnaires (ABPEF and PAQ-A) were administered collectively to secondary education students during school hours, for the school year 2018–2019. After stating the necessary instructions and obtaining the informed consent (from school and families), all the students voluntarily filled out the requested information. The ethical protocols for research were fulfilled, with a special emphasis on confidentiality. The ethical standards established by the Declaration of Helsinki (1975) were followed at all times.

### 2.6. Statistics Analysis

All the analyses were performed with the statistical package SPSS 21.0 [36].

**Figure 1:**
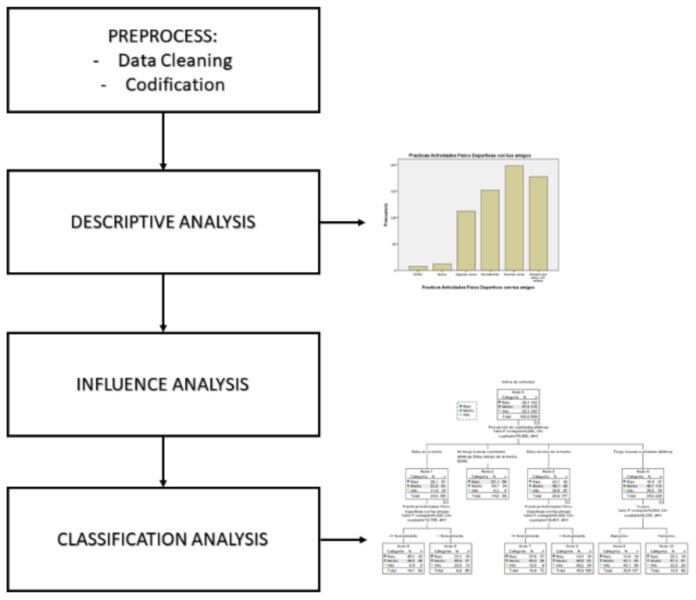
Diagram showing the data analysis process.

After obtaining the data and their digitalization, several statistical analyses were performed in the form of several analysis blocks as shown in Figure 1.

The first step is a descriptive analysis to find the characteristics of the sample as a whole, calculating the mean of position, dispersion and form, so obtaining the first results of homogeneity and the presence of anomalous elements or outliers that can produce incoherent results.

To analyze the effects of influence among the different variables, variance analysis (one-way ANOVA) was performed and correlations analysis using the Spearman’s rho coefficient.

A chi-squared test was performed to check if the qualitative explanatory variables of the model are independent or not of the categorized variable of PA.

In the last stage of the analysis, it is important to point out that the dependent variable, once grouped, has both a quantitative and a qualitative aspect. For that reason, classification and regression trees (CART), using the Chi-square Automatic Interaction Detection (CHAID) algorithm, were used as the optimal techniques due to its capacity to extract conclusions.

The CHAID (Chi-square Automatic Interaction Detection) Segmentation Algorithm, considered a general segmentation algorithm, is used for exploratory and descriptive purposes, with the fundamental objective of finding the partition of a sample of objects into groups capable of best describing the dependent variable. It assumes that the dependent variable is categorical and uses the Chi-square test to test independence at different stages of the process.

The following calculations were performed: error rate as a classification model. Error rate is analyzed in a validation set. Classification rules for each observation. Each observation, subject, enters the model from the top of the tree and follows the downward “path” according to the value of its variables, obtaining in the final node the percentage for each one of the classes. Importance of variables in the prediction for the variable object. According to the position of the variables in the model, higher or lower, an importance is assigned in the classification rule of the model. By the analysis of the percentage of probability of categorizing a subject under a class of PA, the highest and lowest values are analyzed to establish the pattern of the subject with, for instance, the highest probability of sedentariness.

Last, the confidence interval (IC 95%) was determined for the level of PA so it can be determined where the mean level of PA of the whole population of subjects with a set confidence probability occurs.

## 3. Results

Table 2 shows the *p*-values for the two predictor variables of a qualitative nature used as a factor.

Therefore, the analysis demonstrated a statistically significant association between gender and PAQ (F = 24.90; *p*-value = 0.002), as well as between school year and PAQ (F = 30.59; *p*-value = 0.001). However, this does not adjust for the effect of any other variables.

Taking into account the previously mentioned double nature of the variable PA, the level of PA was categorized, generating two qualitative variables segmented, respectively, in three and two categories.

As shown in Table 3, in all cases the variables show an association with the categoric variable PA, matching the ANOVA results, proving the consistency of the propose categorization, keeping the dependency of the explanatory variables both in their quantitative and qualitative aspects.

As seen previously with the variable school year, Table 4 shows the dependency with the category variable PA, matching again the ANOVA results.

Table 5 shows the values found for the correlation coefficient.

It is important to point out that the results show that the values closest to zero are for the variables Environment Barrier and Body Image Barrier, indicating an almost no statistical correlation (Pearson correlation coefficient) with the variable PA, and so have very little influence on it. The factors with a negative correlation, Age and all the barriers, have a negative influence on PA, so an increase in those factors would reduce the level of PA. The factors with the biggest influence, or with the highest correlation, with the variable PA are Age and Tiredness and the Laziness Barrier, with much higher values than the rest of the factors.

**Figure 2:**
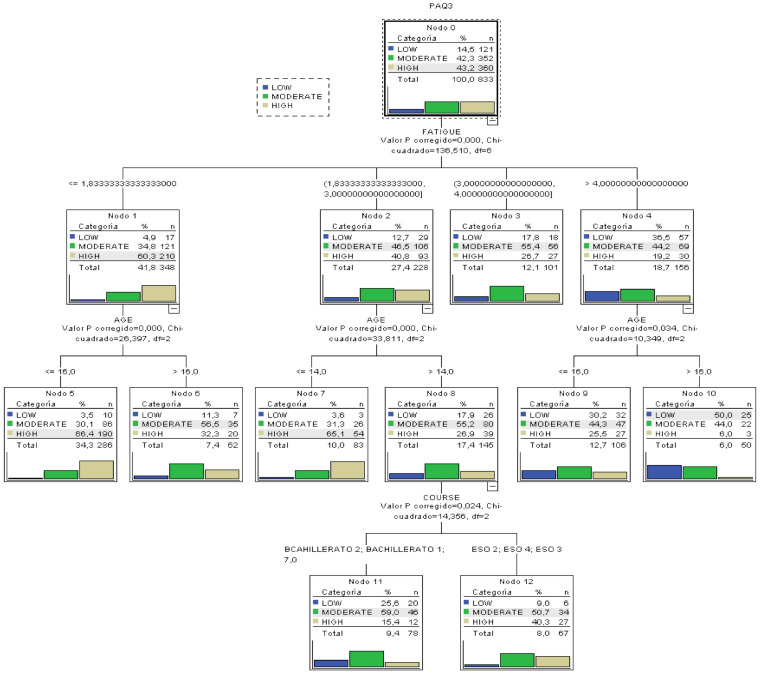
Classification tree for the level of PA categorized as three classes.

As previously described, a classification tree was built with the variable categorized under three classes all with *p*-values lower than 0.001 in all CHAID splits. The resulting tree can be seen in Figure 2.

The principal results after analyzing the tree are:

- The most important variable to categorize the level of PA is the Tiredness and Laziness barrier, because it is higher up in the tree.
- From the ANOVA and correlation analyses (Pearson correlation coefficient) before constructing the tree, the variables with the highest degree of influence in PA had been determined. The tree confirms such analysis because the variables different from age, school year and tiredness barriers do not appear as influential in the tree.
- The most probable case (50%) for a low level of PA can be extracted: a subject with a value in the tiredness and laziness barrier over 4 and over 16 years of age.
- The most probable case of having a high index of PA can be determined: subjects with a value of tiredness barrier over 1.83 and less than 16 years of age present a probability of 66.4% of having a high index of PA.
- Classification rules for the three types of PA can be extracted, besides the most extreme cases previously mentioned.
- In the variable Age, we can see that the threshold value that limits the classification is 16 years, so that is the most important age in the variability of PA.
- In the variable school year, the creation of a group with the oldest students (Baccalaureate) and the youngest (CSE) can clearly be seen. Therefore, in that second group we find a higher probability of having a high index of PA (40%).

**Figure 3:**
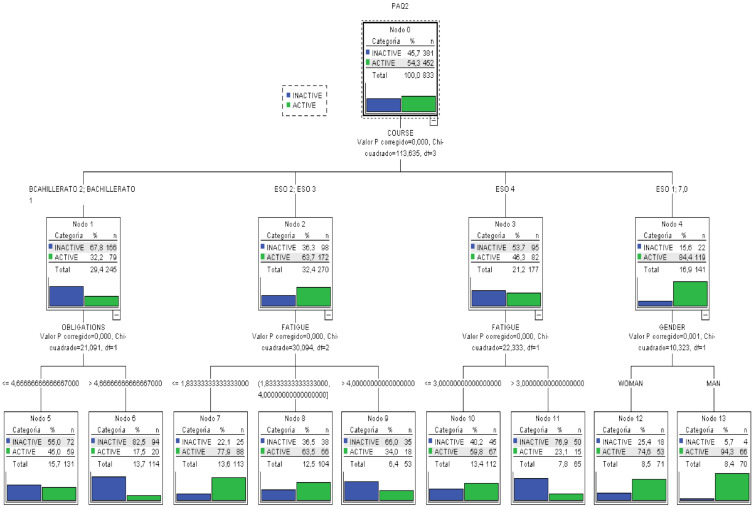
Classification trees for the level of PA categorized into two classes.

Taking into account the latest scientific knowledge, a classification tree with the variable divided into two categories was also created. Figure 3 shows the whole tree.

Under this classification, the main results obtained from analyzing the tree are:

- The most important variable in the categorization of the level of PA is school year, because it is higher up.
- The variables that the ANOVA and correlation analysis had determined not to be influential maintain that status in the model, because they do not appear in any level of the tree.
- The most probable case (82.5%) for a low level of PA can be extracted: a subject in a late school year (Baccalaureate) and many responsibilities.
- The most probable case for a high index of PA can be determined: male subjects in an early school year (CSE 1º) show a probability of 94.3% of having a high index of PA.
- Any classification rules for the three types of PA besides the extreme cases previously discussed.
- In this model, the error rate of the tress as a classification model is 15%, that is, given a new subject, after measuring the different variables, it could be categorized in its level of PA with an error margin of 15%.

The results of both models can be explained because in the first model with three categories, the percentages of each were 14.5% (low), 42.3% (moderate) and 43.2% (high).

In the two categories model, the threshold value for both categories is 2.75, producing a distribution of 54.3% (inactive) and 45.7% (active). This last categorization is more homogeneous by classes, offering a lower error rate than the previous one.

Lastly, the confidence interval (CI) was calculated for the population mean of the level of PA with a confidence of 95% giving the interval (2.8274; 2.9418) with *p* = 0.0001.

## 4. Discussion

This study analyzed the possibility of extracting classification rules for subjects in order to maximize or minimize the probability of having the right level of PA as a function of the explanatory variables, also analyzing the dependencies of those variables. For that purpose, two classification trees were built using the two possible categorizations of the PA variable.

In the model with three classifications, the barrier with most explanatory power on the sedentarism in adolescents is Tiredness and Laziness, especially on those under 16 years of age. The least influential barriers are environment and body image.

However, when using the two categories model, the division between active and inactive participants allows for a better understanding of the results, because in the case of younger children the fundamental difference is school year. In second and third years of CSE, between 13 and 15, the main barrier is Tiredness and Laziness, while in baccalaureate the barrier is time commitments.

Therefore, the perception of barriers preventing the practice of PA is mainly time commitment or lack of time and tiredness or laziness [36,37]. Other studies add also social support, level of energy, motivation, skills, resources and fear of harm during the activity [7]. Those in a low socioeconomic level suffer a higher risk of perceiving barriers such as a lack of motivation and lack of resources. Parents with low incomes mention significantly higher barriers [14]. Community factors are often mentioned as barriers, especially those related to the built environment [14].

It must be mentioned that the adolescents do not indicate the environment and the facilities as barriers, contrary to adults. In the case of older adults, the environment (connectivity of the streets, walking facilities, aesthetics, road safety and personal safety) predicts the increase in the time walking [38]. It seems that each group, according to age, has specific barriers. Furthermore, variables such as socioeconomic level, age, gender, marital status, education level and self-perception of health are factors that also predict the barriers for PA [7,39]. Individuals of low socioeconomic status are at greater risk of perceiving barriers such as lack of motivation and lack of resources. However, individuals with partners do not perceive lack of social support and lack of motivation as barriers to PA [7]. Low-income parents reported significantly higher barriers [14].

Taking into account the CI, we can estimate with a high probability that the level of PA in children between 12 and 18 years is moderate, because the range of values is small, and the level of physical activity is situated at a score of around three.

The results match the scientific evidence from the studies performed in recent years. Children and adolescent are increasingly performing more sedentary activities. In fact, they are frequent users of television [40,41]. In relation to the beliefs that guide the decisions of the parents, Hamilton et al. [42] point out as barriers to the practice of PA the lack of time and fatigue of the parents, and as facilitating variables the access to parks, the social support to be able to involve your child in an adequate PA and control the time for which they are using data visualization screens (PVD).

Moreover, adolescents manifest a delay of the circadian rhythm, so they have problems falling asleep [43]. It is increasingly habitual for them to stay up very late using their cell phones [44,45], so they do not reach the 7.5 h of recommended sleep [46]. Such circadian changes can have a negative impact on their vitality and their school performance [47]. Furthermore, the pressure they suffer at school makes them devote much time to those tasks, tiring them. Therefore, consequently, the increase in tiredness also increases their levels of laziness.

## 5. Limitations of the Study

Evidently, the results can only be applied in the context of countries with a development level similar to the Galician socioeconomic context, because the reported barriers can be different to those found in other places [48].

Another limitation of the study is not taking into account factors such as the socioeconomic level of the family or the education level of the parents, factors predicting barriers for PA as made clear in the theoretical framework [7].

Furthermore, this analysis did not account for the nested data structure inherent in the study design, given the similarity of the contexts of the centers included in the sample.

## 6. Conclusions

We can conclude that the barriers explaining the level of PA in adolescents are tiredness and laziness and time commitments after 16 years of age. In fact, as the students advance through school, that is, their age increases, the level of PA and the barriers most influential in the model are different, standing out responsibilities and commitments. In all cases, the least influential barriers in the practice of PA are the environment and body image, and the most determining of the level of PA is the age of the subject. Furthermore, we can attest that the index of PA is moderate.

Sedentary behaviors, physical inactivity and BMI among children and adolescents are some of the main public health problems. The knowledge of which barriers determine the practice of PA is a very important variable in the creation of action plans and social policies to solve that problem.

To avoid the barriers and prevent obesity, as pointed out by the WHO (World Health Organization) [49], it would be of the upmost importance to have favorable environments and communities that can influence the choices of the individual in such a way that the simplest option (that is, the easier to access, use and acquire) is the healthiest in terms of food and habitual PA. Even though the environment is not a barrier among young people, it would also be important to analyze the design of cities and environments, and the size of the population due to its influence in the individual decisions causing laziness and apathy in the subjects. In addition, awareness programs are needed to improve parents’ perception of their children’s weight so that they can engage in physical activities that can prevent their obesity [5].

As in adolescence the level of tiredness is highest, it is advisable to also stress the need to sleep the right number of hours (between 8 and 10), adopt a rich and varied diet and regularly practice a sport offering not only physical but also emotional wellbeing. It is recommended to start that process at a very early age, with the support of the family to consolidate the habits as quickly as possible. The school, as an agent of socialization and human values, must also become the facilitator of an active city collaborating in the transformation toward habits of PA or sport.

## Figures and Tables

**Table 1:** Frequency and percentages for the variable school year.

	CSE 1	CSE 2	CSE 3	CSE 4	BAC 1	BAC 2	Total
Frequency	140	130	140	177	151	95	833
Percentage	16.8	15.6	16.8	21.2	18.1	11.4	100.0

**Table 2:** ANOVA results of the level of practice of physical activity (PA) according to V.I.

Variable	Category	N	Mean PA	F	*p*-Value
Gender	Man	407	3.031	24.90	0.002
	Woman	426	2.744		
School year	CSE 1	140	3.402	30.59	0.001
	CSE 2	130	3.131		
	CSE 3	140	2.993		
	CSE 4	177	2.738		
	BAC 1	151	2.614		
	BAC 2	95	2.323		

Legend: Compulsory Secondary Education primero (CSE1) and the following courses and Baccalaureate primero (BAC 1) and the following course.

**Table 3:** Chi-squared test of dependency of the variable school year on the Physical Activity Questionnaire (PAQ).

Chi-Square = 130.943; Sign. = 0.002			PAQ			Total
			Low	Moderate	High	
SCHOOL YEAR	CSE 1	Count	5	35	100	140
		% of SCHOOL YEAR	3.6%	25.0%	71.4%	100.0%
	CSE 2	Count	11	45	74	130
		% of SCHOOL YEAR	8.5%	34.6%	56.9%	100.0%
	CSE 3	Count	14	55	71	140
		% of SCHOOL YEAR	10.0%	39.3%	50.7%	100.0%
	CSE 4	Count	31	85	61	177
		% of SCHOOL YEAR	17.5%	48.0%	34.5%	100.0%
	BAC 1	Count	29	78	44	151
		% of SCHOOL YEAR	19.2%	51.7%	29.1%	100.0%
	BAC 2	Count	31	54	10	95
		% of SCHOOL YEAR	32.6%	56.8%	10.5%	100.0%
	Total	Count	121	352	360	833
		% of SCHOOL YEAR	14.5%	42.3%	43.2%	100.0%

Legend: Compulsory Secondary Education primero (CSE1) and the following courses and Baccalaureate primero (BAC 1) and the following course.

**Table 4:** Chi-squared test of the dependency of gender on PAQ.

Chi-Squared = 26.193; Sign. = 0.001			PAQ			Total
			Low	Moderate	High	
Gender	Man	Count	46	149	212	407
		% for Gender	11.3%	36.6%	52.1%	100.0%
	Woman	Count	75	203	148	426
		% for Gender	17.6%	47.7%	34.7%	100.0%
Total		Count	121	352	360	833
		% for Gender	14.5%	42.3%	43.2%	100.0%

**Table 5:** Pearson’s correlation coefficient and level of significance between the quantitative independent variables and the index of PA.

Variable	Correlation Coeff.	*p*-Value
Age	−0.388	0.001
BMI	0.0049	0.887
Environment Barrier	−0.0963	0.005
Tiredness and Laziness Barrier	−0.3897	0.002
Body Image Barrier	−0.1446	0.001
Responsibilities Barrier	−0.2577	0.001
